# The performance of anthropometric tools to determine obesity: a systematic review and meta-analysis

**DOI:** 10.1038/s41598-020-69498-7

**Published:** 2020-07-29

**Authors:** Isolde Sommer, Birgit Teufer, Monika Szelag, Barbara Nussbaumer-Streit, Viktoria Titscher, Irma Klerings, Gerald Gartlehner

**Affiliations:** 1 Department for Evidence-Based Medicine and Evaluation, Danube University Krems, Dr.-Karl-Dorrek-Straße 30, 3500 Krems, Austria; 2 RTI-UNC Evidence-Based Practice Center, Research Triangle Institute International, East Cornwallis Road, Post Office Box 12194, Research Triangle Park, NC 27709-2194 USA

**Keywords:** Disease prevention, Weight management

## Abstract

The aim of this systematic review was to assess the performance of anthropometric tools to determine obesity in the general population (CRD42018086888). Our review included 32 studies. To detect obesity with body mass index (BMI), the meta-analyses rendered a sensitivity of 51.4% (95% CI 38.5–64.2%) and a specificity of 95.4% (95% CI 90.7–97.8%) in women, and 49.6% (95% CI 34.8–64.5%) and 97.3% (95% CI 92.1–99.1%), respectively, in men. For waist circumference (WC), the summary estimates for the sensitivity were 62.4% (95% CI 49.2–73.9%) and 88.1% for the specificity (95% CI 77.0–94.2%) in men, and 57.0% (95% CI 32.2–79.0%) and 94.8% (95% CI 85.8–98.2%), respectively, in women. The data were insufficient to pool the results for waist-to-hip ratio (WHR) and waist-to-height ratio (WHtR) but were similar to BMI and WC. In conclusion, BMI and WC have serious limitations for use as obesity screening tools in clinical practice despite their widespread use. No evidence supports that WHR and WHtR are more suitable than BMI or WC to assess body fat. However, due to the lack of more accurate and feasible alternatives, BMI and WC might still have a role as initial tools for assessing individuals for excess adiposity until new evidence emerges.

## Introduction

Obesity is widely recognised as a pandemic public health problem. According to the World Health Organization (WHO), in 2016 more than 650 million adults worldwide were obese^1^. These numbers have almost tripled since 1975^2^. Obesity increases the risk for many chronic diseases, such as diabetes mellitus, cardiovascular diseases and cancers^3^, and is possibly associated with mental health disorders^4^. Associations have been shown to be strongest between obesity and the incidence of diabetes mellitus, particularly in women (risk ratio [RR] 12.41, 95% confidence interval [CI] 9.03–17.06).

Primary care is considered one of the main settings for the prevention, screening and management of obesity^5^. Individual studies indicate that patients are more likely to lose weight when they receive recommendations for lifestyle changes from their primary care physicians^6^. Because it can be difficult for physicians to accurately determine obesity solely through visually inspecting their patients^7^, they need a reliable, efficient screening tool in order to ensure that those who need management and treatment receive it.

WHO conceptualises obesity as “abnormal or excessive fat accumulation that may impair health”^1^. It is most commonly assessed using body mass index (BMI), a simple and quick anthropometric tool that has a low cost. Adults with a BMI greater than or equal to 30 are classified as being obese^1^ (Table 1). However, several researchers and professional associations^8–14^ consider the use of BMI as the primary clinical index of obesity insufficient. They have called for a new definition that fully accounts for the complexity of the disease relating to the quantity, distribution and secretory function of adipose tissue.

**Table 1:** Definitions of anthropometric measurement tools.

*Body mass index (BMI)*: person's weight in kilograms divided by the square of his/her height in meters (kg/m^2^)
*Waist circumference (WC)*: waist circumference measured at approximate midpoint between the lowest rib and the top of the iliac crest
*Waist-to -hip ratio (WHR)*: person’s waist measurement divided by hip measurement taken around the widest portion of the buttocks
*Waist-to-height ratio (WHtR)*: person’s waist circumference divided by their height

*Source*: World Health Organization^85^.

A substantial body of evidence has shown that obesity (BMI ≥ 30) is associated with an increased risk of coronary heart disease^15^ and mortality^16^ relative to normal weight. For mortality, this association follows a J-shaped curve. Although a significantly higher mortality rate was found for all obesity grades combined (hazard ratio [HR] 1.18 [95% CI 1.12–1.25]), being overweight (BMI of 25–< 30) reduced the risk of all-cause mortality (0.94 [95% CI 0.91–0.96]), and grade 1 obesity (BMI of 30–< 35) was not related with higher mortality (0.95 [95% CI 0.88–1.01])^16^. In older age, overweight and obesity as defined by BMI might even be protective against mortality^17–19^.

Indeed, one of the main deficiencies of BMI is that it does not differentiate between fat mass and fat-free mass. Not all people with high levels of body fat have a BMI of 30 or greater, and some people with very high BMIs may have little fat mass. The proportion of body fat also differs across ethnic populations, sex, and age groups. For example, South Asian populations have a higher proportion of body fat than Caucasians for the same BMI^20^. Women have a significantly higher percentage of total and sub-cutaneous fat stores than their male counterparts^21^. The proportion of internal fat increases and muscle mass decreases with age, which can lead to sarcopenic obesity, the combination of obesity and muscle impairment^22^. In older populations, research even suggests that fat mass is associated with a decreased risk of morbidity and mortality^17,19,23,24^, while a low fat-free mass might be a risk factor for mortality^25,26^.

Another main deficiency of BMI is that it does not account for body fat distribution. The distribution of body fat is associated with the risk of metabolic syndrome and other cardiometabolic complications^10^. Longitudinal data have shown that the distribution of excess fat (resulting in a so-called apple or pear shape) has a greater influence on certain health risks, such as cardiovascular diseases or cancer, than total body fat^27,28^. Indices assessing the distribution of body fat include waist circumference (WC), waist-to-hip ratio (WHR) or waist-to-height ratio (WHtR) (Table 1). A growing body of evidence suggests that such indices are independently associated with cardiometabolic diseases and mortality^29–31^. They could thus provide additional value in determining obesity and the risk for associated comorbidities in clinical practice.

Imaging techniques allow for the measurement of body fat, its distribution, and body composition but are rarely used in clinical practice. They are generally considered more precise than anthropometric methods and continue to serve as “reference standards” in many research studies^14^ until the concept of obesity is fully understood.

Despite the definitional problems with BMI, it remains the routine measurement to classify obesity in clinical practice. Within the last two decades, only two systematic reviews on the performance of anthropometric tools compared to that of body composition techniques have been published. The review by Okorodudu et al.^32^ focused on the performance of BMI, and Mc Tigue et al.^33^ reviewed the performance of BMI, WC and WHR in older adults. Both reviews are relatively old, with Okorodudu et al.^32^ searching for studies until June 2008 and Mc Tigue et al.^33^ until February 2003. Due to the emergence of new research evidence and the development of anthropometric tools other than BMI, we have aimed to provide an up-to-date systematic review using four anthropometric tools (BMI, WC, WHR and WHtR) for determining obesity in the adult population.

## Methods

This systematic review was conducted following the Cochrane Methods for Systematic Reviews of Diagnostic Test Accuracy^34^ and reported according to the Preferred Reporting Items for a Systematic Review and Meta-Analysis of Diagnostic Test Accuracy Studies (PRISMA-DTA) statement^35^. The protocol is registered with the International Prospective Register of Systematic Reviews (PROSPERO), registration number CRD42018086888.

### Information sources and searches

We searched the electronic databases Ovid MEDLINE, Embase.com (Elsevier), CINAHL (Ebsco) and PubMed (non-MEDLINE content) from 1 January 2000 to 16 January 2018, as well as the dissertation databases ProQuest Dissertations & Theses Global (ProQuest) and WorldCat dissertations from 1 January 2000 to 16 January 2018. In addition, we manually searched the reference lists of recent and relevant systematic reviews. Searches were limited to English and German language documents. An experienced information specialist developed a search strategy for Ovid/Medline MEDLINE, amended it to fit other electronic databases and performed all searches (see Supplementary file 1). In line with the peer review of the electronic search strategy (PRESS) statement^36^, the Ovid MEDLINE search strategy was peer-reviewed by another information specialist.

### Inclusion criteria

We included randomised controlled trials and prospective cohort or cross-sectional diagnostic studies assessing the performance of anthropometric tools (BMI, WC, WHR and WHtR) to determine obesity in adults (≥ 18 years) from any country. Our target population was adults aged 18 years from any country. We did not exclude studies with adults with diseases or disabilities that could have an impact on the body fat distribution. We used imaging techniques including computed tomography (CT), magnetic resonance imaging (MRI), dual energy X-ray absorptiometry (DXA) and ultrasound scanning (US) as reference standards because they are currently considered the most precise methods for assessing body composition^37^. We included studies that reported sensitivity, specificity, predictive values, likelihood ratios, diagnostic odds ratios, positivity thresholds or receiver operating characteristics (ROC) curves (including area under the curve [AUC]) as outcomes. The eligibility criteria are described in more detail in Table 2.

**Table 2:** Eligibility criteria studies relevant to this systematic review.

Characteristics	Inclusion	Exclusion
Population	Adults aged 18 years and above	Children, adolescents, and pregnant women
Index tests	Body mass index, waist circumference, waist-to-height ratio, waist-to-hip ratio	Any other anthropometric test, non-anthropometric tests
Target conditions	Overall and abdominal overweight, obesity, body fat distribution	Other conditions
Reference test	Imaging techniques including computed tomography, magnetic resonance imaging, dual energy X-ray absorptiometry, and ultrasound scanning	Any other tests measuring body composition including body volume/density tests, total body water or hydrometry tests, and impedance analyses
Outcomes	Sensitivity, specificity, predictive values, likelihood ratios, diagnostic odds ratios, positivity thresholds, ROC curves (including AUC)	Agreement measures
Study design	Randomised controlled trials (RCTs), prospective cohort studies, and cross sectional studies	All other study designs
Setting	Any setting	
Publication date	1/2000 onwards	Before 1/2000
Publication language	English, German	Other languages

### Study selection

**Figure 1:**
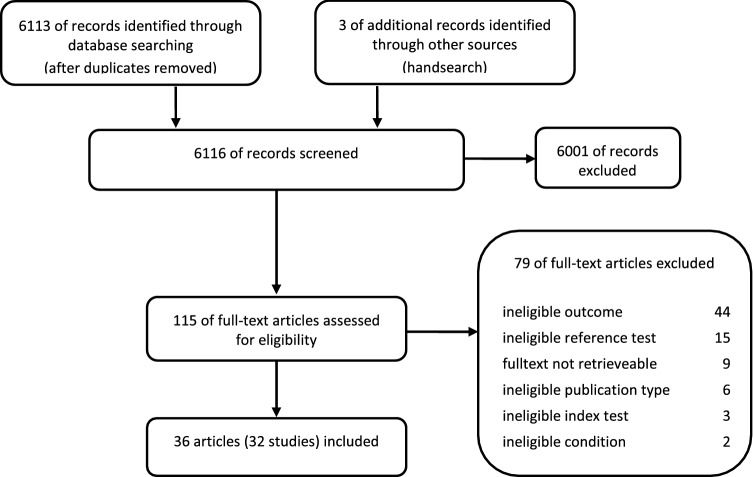
Flow chart of study selection process.

We developed and pilot-tested abstract and full-text review forms that reflected our inclusion and exclusion criteria. Two reviewers independently screened abstract and full-text articles and evaluated their eligibility for inclusion. Any discrepancies were resolved through discussion and consensus or by consultation with a third reviewer. The abstract and full-text reviews were carried out with Covidence (https://www.covidence.org/). Figure 1 summarises the flow of the literature review.

### Data collection process and data items

We designed and pilot-tested a structured data abstraction form. One reviewer extracted data, and another checked for completeness and accuracy. For studies that met our inclusion criteria, we abstracted information related to (a) population; (b) index tests; (c) reference test; (d) obesity; (e) diagnostic values and f) funding source. We extracted or reconstructed the original classification data (2 × 2 table) at or close to WHO’s recommended cut-offs (BMI: ≥ 30 kg/m^2^, WC: ≥ 88 cm in women and ≥ 102 cm in men, WHR: 0.85 in women and 0.90 in men)^38^ or utilised common definitions (body fat percentage: > 35% in women and > 25% in men) for further use in the meta-analyses. Otherwise, definitions of obesity as laid out in the articles were used. We contacted study authors via email if relevant data were not reported in an included publication.

### Risk of bias and certainty of evidence assessment

Two independent reviewers assessed the risk of bias of diagnostic accuracy studies using the Quality Assessment of Diagnostic Accuracy Studies (QUADAS-2) tool^39^. We dually assessed the certainty of evidence for relevant outcomes using the Grading of Recommendations Assessment, Development and Evaluation (GRADE) approach for diagnostic tests^40^. We resolved disagreements by discussion and consensus or by consulting a third reviewer.

### Data synthesis

We conducted meta-analyses using the metandi command in STATA (version 15, Stata Corp.) when five or more studies were similar in terms of the index test, target condition and cut-offs used. The metandi command uses hierarchical logistic regression models to calculate meta-analyses of pairs of sensitivities and specificities. It displays the pooled estimates in both a bivariate and a hierarchical summary receiver operating characteristics (HSROC) model^41,42^. For each index test, we produced a paired forest plot of each study’s sensitivity and specificity, as well as a plot of the sensitivities versus specificities in the ROC space. We assessed the heterogeneity by visually inspecting the CIs for sensitivity and specificity in the paired forest plots. For those index tests where we did not have sufficient studies to pool, we synthesised the data narratively.

Because of differences in the definition of the target condition between men and women, we conducted all analyses separately by sex. When information was available, we analysed the data by ethnicity. We further conducted sensitivity analyses to determine the impact of study quality on the robustness of the overall test performance measures. Subgroup analyses by age were not possible due to dissimilarities in the age categories in the studies.

## Results

Our search yielded 6,116 records of which 32 studies (reported in 36 publications) met our a priori–defined eligibility criteria (Fig. 1)^43–78^. Twenty-seven studies (29 articles) assessed BMI^44,46–54,58–61,64–78^, and 15 (19 articles) reported on waist measurements such as WC^43–47,49–53,57–60,62,63^, WHR^43–47,49–51^ and WHtR^51–56^.

The majority of studies used DXA to evaluate anthropometric measurement tools^44,46,48,50–54,58–78^, while four studies used CT^43,45,55–57,68^ and three used MRI^46,47,49^. The cut-offs for obesity with DXA ranged from ≥ 30% to ≥ 43% body fat in women and from ≥ 20 to ≥ 34.6% in men. Studies using CT or MRI applied a cut-off of ≥ 100 cm^2^ or ≥ 130 cm^2^ of visceral adipose tissue area to provide reference data for both women and men. Fourteen studies were community-based^44,46,51,58,62–64,66–68,70,73,75,76,78^, fourteen were primary care– or hospital-based^45,47,49,50,52–54,57,59–61,69,72,74,77^, two were community- and hospital-based^43,48,55,56^, one study was based in the army^65^ and one did not report its setting^71^. Six studies included patients with various diseases or physical or cognitive disabilities^50,52–54,59,61,66,74^. Four studies stratified analyses by age groups^43,44,46,55,56,58,78^. The prevalence of obesity ranged widely from 5.7^51^ to 95.8%^67^. Four studies did not report any prevalence data^45,47,60,73^.

Of the included studies, we rated the risk of bias for six as low^52,53,58,60,63,67,70^, for 16 as unclear^43–47,54–57,59,62,65,66,69,71,73–75,77^ and for ten as high^48–51,61,64,68,72,76,78^. The reasons for the high risk of bias ratings included convenience sampling, inappropriate exclusion criteria for study participants and lack of predefined thresholds for index and reference tests. Eleven studies were conducted in Asia^44,46,47,49,51,60,63,68,70,71,75,78^, ten studies in North America^50,52–54,58,65,66,72–74,77^, eight in South America^43,45,55–57,59,61,62,67,69^ and three^48,64,76^ in Europe. Twenty-two studies were publicly funded, eight studies did not report their funding^48,49,64,65,68,69,71,76^ and two received sponsoring from pharmaceutical companies^57,61^. Supplementary file 2 (Table S1) presents the characteristics of the included studies.

In the following sections, we first present the results of the four anthropometric measurement tools for women and men separately and if data allow, stratified by different ethnicities.

### Body mass index (BMI)

The 27 included studies^44,46–54,58–61,64–78^ reported varying cut-offs for BMI to determine obesity. Thresholds ranged from 19.6 to 30 kg/m^2^ for women and from 23.5 to 30 kg/m^2^ for men. Studies often applied more than one cut-off to identify the threshold with the highest discriminative power. Cut-offs applied to Asian populations were generally lower than in other populations. Around 75% of studies from various countries, however, used the cut-off for obesity as suggested by WHO (≥ 30)^1^ (see the characteristics of the included studies in Supplementary file 2, Table S1 and the results of all studies in Supplementary file 3, Table S1).

**Figure 2:**
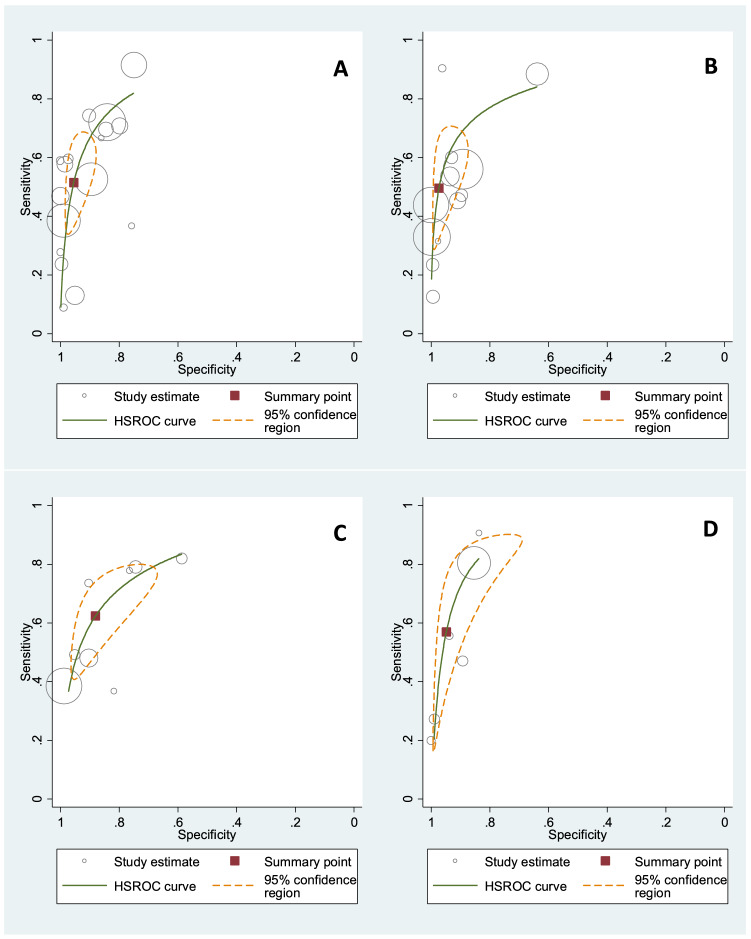
Summary ROC curve including summary point for Body Mass Index (BMI) in (**A**) Women (sensitivity: 51.4% [95%CI 38.5–64.2%]; specificity: 95.4% [95%CI 90.7–97.8%]) (**B**) Men (sensitivity: 49.6% [95% CI 34.8–64.5%]; specificity: 97.3% [95% CI 92.1–99.1%]) and for Waist Circumference (WC) in (**C**) Women (sensitivity: 62.4% [95% CI 49.2–73.9%] and for specificity 88.1% [95% CI 77.0–94.2%]) (**D**) men (sensitivity: 57.0% [95% CI 32.2–79.0] and for specificity 94.8% [95% CI 85.8–98.2%]).

**Figure 3:**
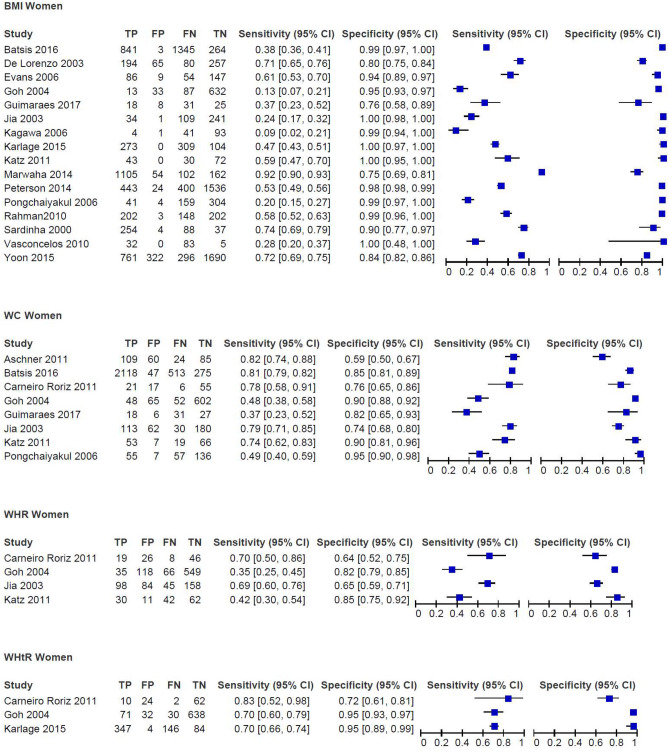
Paired forest-plots of sensitivity and specificity for body mass index (BMI), waist circumference (WC), waist-to hip ratio (WHR), waist to height ratio (WHtR) in women; data not weighted; TP, true positives; FP, false positives, FN, false negatives; TN, true negatives.

Based on a meta-analysis of 16 studies with data on 14,008 women of any race or ethnicity, the combined sensitivity of BMI (with thresholds from 25 to 30 kg/m^2^) to detect obesity was 51.4% (95% CI 38.5–64.2%), with a corresponding specificity of 95.4% (95% CI 90.7–97.8%), as shown in the HSROC plot (Fig. 2). The HSROC plot shows the individual study estimates, a summary curve from the HSROC model, a summary estimate, a 95% confidence region for the summary estimate and a 95% prediction region. The confidence intervals of some studies failed to overlap for sensitivity, indicating considerable heterogeneity. The heterogeneity of the specificity was low (Fig. 3). A sensitivity analysis excluding studies with a high risk of bias had little impact on the results (sensitivity: 48.0% [95% CI 30.6–67.4%] and specificity: 96.1% [95% CI 88.3–98.8%]). When we excluded studies in Asian women from the meta-analyses, all the remaining studies (10 studies, n = 7,640) used a BMI cut-off of 30 kg/m^2^. The results of the meta-analysis focusing on White, Latin or women of mixed ethnicity neither substantially altered the heterogeneity nor the summary estimates of the meta-analysis (sensitivity: 52.9% [95% CI 43.8–61.9%] and specificity: 97.0% [95% CI 90.8–99.1%]) (see Supplementary file 4, Figures S1 and S2). We rated the certainty of evidence of the pooled studies and considered it as very low for the sensitivity and as moderate for the specificity. The reasons for downgrading the certainty of evidence included the wide range and confidence intervals of the results for the sensitivity and the risk of bias for the specificity.

**Figure 4:**
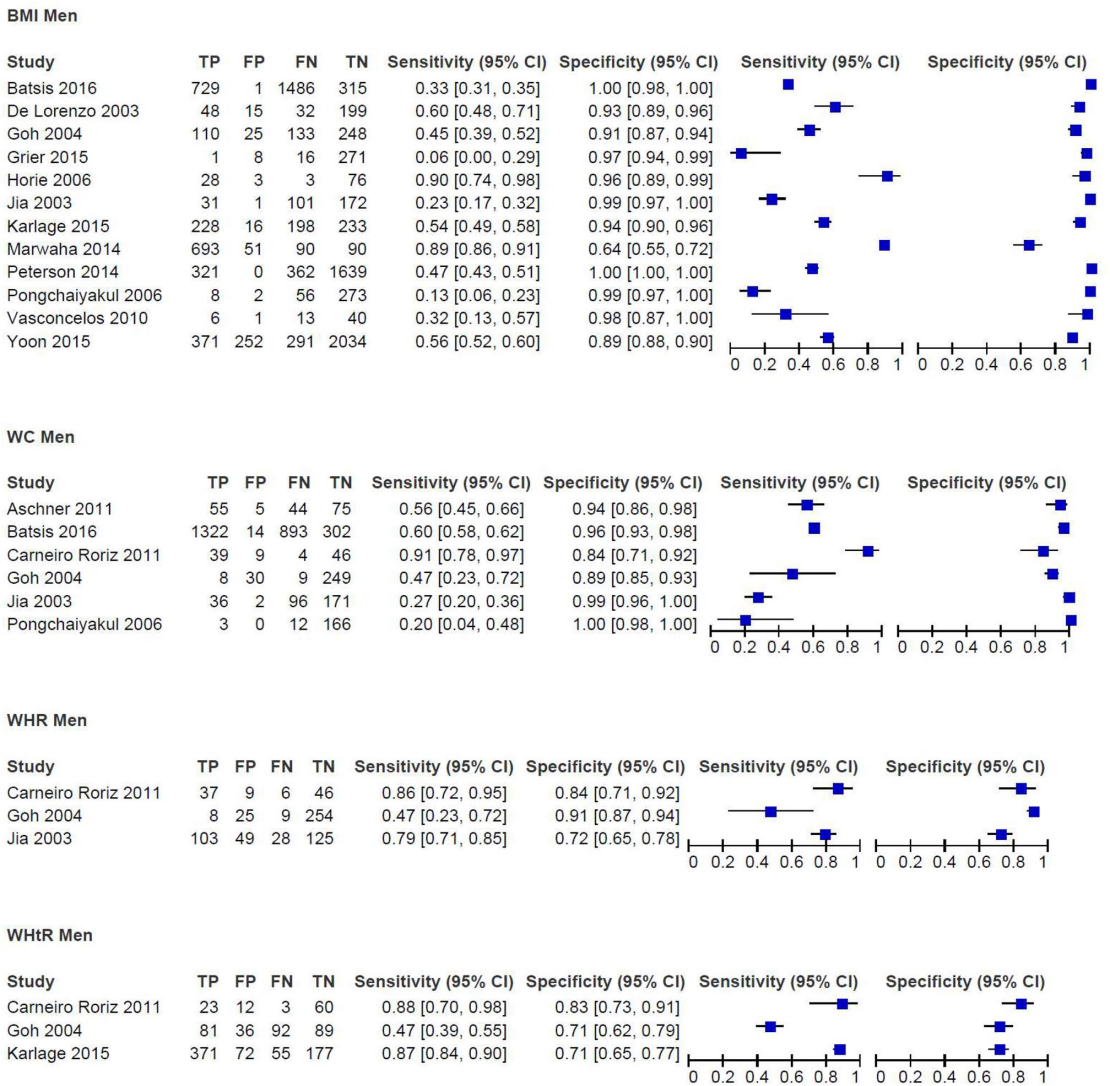
Paired forest-plots of sensitivity and specificity for body mass index (BMI), waist circumference (WC), waist-to hip ratio (WHR), waist to height ratio (WHtR) in men; data not weighted; TP, true positives; FP, false positives, FN, false negatives; TN, true negatives.

In men, the results of a meta-analysis including 12 studies with data on 11,320 men of any race or ethnicity show a combined sensitivity of 49.6% (95% CI 34.8–64.5%) and a specificity of 97.3% (95% CI 92.1–99.1%) for BMI cut-offs from 25 to 30 kg/m^2^ (Fig. 2). The sensitivity varied considerably across studies (Fig. 4). A sensitivity analysis excluding studies with a high risk of bias had little impact on the results (52.4% [95% CI 28.6–75.1%] and specificity: 98.6% [95% CI 92.2–99.8%]). A subgroup analysis that excluded studies on Asian men and focused on men of White, Latin or mixed ethnicity (6 studies, n = 5,991, cuts-offs: 28.5–30 kg/m^2^) had little effect (sensitivity: 52.8% [95% CI 36.4–68.6%] and specificity: 98.9% [95% CI 93.8–99.8%]; see Supplementary file 4, Figures S1 and S2). We considered the certainty of evidence as very low for the sensitivity and moderate for the specificity. The reasons for downgrading the certainty of evidence included risk of bias as well as the wide range and confidence intervals of the sensitivity results.

### Waist circumference (WC)

For WC, the cut-offs to determine obesity in all 14 included studies^43–47,49–53,57–60,62,63^ ranged from 65.8 to 107 cm in women and from 78.9 to 105 cm in men. Similar to studies assessing the performance of BMI, the analyses often applied more than one cut-off (see the characteristics of the included studies in Supplementary file 2, Table S1 and the results of all studies in Supplementary file 3, Table S2).

A meta-analysis including eight studies on 4,964 women rendered a sensitivity of 62.4% (95% CI 49.2–73.9%) and a specificity of 88.1% (95% CI 77.0–94.2%) for WC (80.5 to 92.3 cm) (Fig. 2). For both sensitivity and sensitivity, the heterogeneity of the included studies was high (Fig. 3). Excluding the study by Goh et al.^51^ from the analysis because of its low cut-off (80.5 cm) did not substantially alter the results (cut-offs 86 cm to 92.3 cm; sensitivity: 64.4% [95% CI 50.1–76.6%] and specificity 88.0% [95% CI 74.5–94.8%]). Likewise, the results remained similar when excluding studies with a high risk of bias^49–51^ (cut-offs 86–92.3 cm; sensitivity: 58.6% [95% CI 41.0–74.3%] and specificity 89.4% [95% CI 71.1–96.6]). Subgroup analysis on Latin women or women with mixed ethnicity (5 studies, n = 3,557, cuts-offs: 86–92.3 cm) reduced the heterogeneity and increased the sensitivity (73.4% [95% CI 52.5–87.4%]) but decreased the specificity (83.0% [95% CI 62.7–93.4%]; see Supplementary file 4, Figures S1 and S2). Because of methodological concerns and highly inconsistent and heterogeneous results, we rated the certainty of evidence as very low for both sensitivity and specificity.

In men, the pooled estimates of six studies including 3,590 male participants were 57.0% (95% CI 32.2–79.0) for the sensitivity and 94.8% (95% CI 85.8–98.2%) for the specificity (Fig. 2). The cut-offs for WC ranged from 90.2 to 100.0 cm. The results of the included studies had a high heterogeneity for the sensitivity and a low heterogeneity for the specificity (Fig. 4). We were not able to perform sensitivity analyses or subgroup analysis due to the low number of studies included in the meta-analyses. Due to serious methodological concerns and highly inconsistent and heterogeneous results, we considered the certainty of evidence for the sensitivity as very low and for the specificity as low.

### Waist-to-hip ratio (WHR)

The cut-offs for determining obesity in the seven studies reporting on WHR ranged from 0.74 to 0.97 in women and from 0.85 to 0.96 in men^43–47,49–51^ (see the characteristics of the included studies in Supplementary file 2, Table S1 and the results of all studies in Supplementary file 3, Table S3). We did not have enough data to conduct meta-analyses, as only four studies provided 2 × 2 tables^43,49–51^. In women, the sensitivities for WHR ranged from 34.4^51^ to 92.3%^47^, while the specificities ranged from 45.7^47^ to 85.0%^51^ (Fig. 3 and Supplementary file 3, Table S3). Two studies analysed the performance of WHR by age groups. Carneiro Roriz et al.^43^ found sensitivity and specificity to be highest in young and middle-aged women (21–59 years) in their study (n = 99) (sensitivity 0.83, specificity 0.72, no test for interaction). The study by Yang et al. and Li et al.^44,46^ (n = 879) reported a higher sensitivity but a lower specificity in the 20–30 year-old age group compared to that in 31–45 year-old women (sensitivity 0.74 vs. 0.69, specificity 0.65 vs. 0.79, no test for interaction).

In men, the sensitivity ranged from 46.7^51^ to 88.9%^44,46^, and the specificity ranged from 25.0^47^ to 90.9%^51^ (Fig. 4). When stratifying the analysis by age groups, Carneiro Roriz et al.^43^ found similar results in young and middle-aged men (21–59 years, n = 51) and elderly men (≥ 60 years, n = 47) (sensitivity 86.7% vs. 86.2%, specificity 83.3% vs. 83.3%, no test for interaction). Yang et al. and Li et al.^44,46^ reported a similar sensitivity but a lower specificity in 31–45 year-old men (n = 185) compared to that in 20–30 year-old men (n = 694) (sensitivity 88.9% vs. 82.4%, specificity 64.1% vs. 78.4%, no test for interaction).

We rated the certainty of evidence for the sensitivity and specificity as very low in women and in men. The reasons for downgrading the certainty of the evidence related to methodological concerns, heterogeneous results and wide confidence intervals.

### Waist-to-height ratio (WHtR)

We identified four studies^51–56^ assessing WHtR. The data were insufficient to combine the results in a meta-analysis. Their cut-offs for defining obesity ranged from 0.50 to 0.59 in women and from 0.50 to 0.55 in men (see the characteristics of the included studies in Supplementary file 2, Table S1 and the results of all studies in Supplementary file 3, Table S4). In women, the sensitivity ranged from 51.0%^51^ to 83.3%^55,56^ and the specificity from 78.6%^55,56^ to 95.2%^54^ (Fig. 3 and Supplementary file 3, Table S4). The results in men were similar, from 46.7^51^ to 86.7%^54–56^ for the sensitivity and from 71.0^54^ to 89.4%^51^ for the specificity (Fig. 4 and Supplementary file 3, Table S4). Carneiro Roriz et al.^55,56^ did not identify any differences in sensitivity and specificity between adults aged 20 to 59 years and adults aged 60 and older, irrespective of sex. Oreopoulos et al.^52,53^ used slightly higher cut-offs for defining obesity (0.615 for women and 0.605 for men) in their study and reported a combined sensitivity of 77.4% and a specificity of 76.9%.

We rated the certainty of evidence for the sensitivity and specificity in both women and men as very low. The reasons for downgrading the certainty of evidence included methodological concerns, heterogeneous results and wide confidence intervals of the results.

## Discussion

To the best of our knowledge, our work is the most recent and most comprehensive systematic review on the use of four anthropometric tools to determine obesity. Our findings, in general, indicate a lack of reliable scientific evidence on the performance of anthropometric tools to rule out or determine obesity as assessed by imaging techniques, which constitute the gold standard in obesity research until the concept of obesity is fully understood. Many of the included studies were fraught with methodological shortcomings. Consequently, we rated the certainty of evidence as low or very low, which indicates that we have little or very little confidence in the estimates of the effects.

The available studies focused mainly on BMI and WC to assess obesity. The pooled results of our meta-analyses consistently rendered low sensitivities and relatively high specificities for BMI and WC when compared to imaging techniques as reference standards. The sensitivities ranged from 49.6% (BMI for men) and 51.4% (BMI for women) to 57.0% (WC for men) and 62.4% (WC for women) in the pooled analyses. By contrast, the specificities ranged from 88.1% (WC for women) and 94.8% (WC for men) to 95.4% (BMI for women) and 97.3% (BMI for men).

These estimates are consistent with the findings from a previous systematic review by Okorodudu et al.^32^, who reported a pooled sensitivity of 50% (95% CI 43–57%) and a pooled specificity of 90% (95% CI 86–94%) in their review of 25 studies. The studies included in this review went back to the 1990s and also used reference standards other than imaging techniques. For our systematic review, we employed more rigorous eligibility criteria than the Okordudu review^32^ and included 17 additional studies that were published after the literature searches by Okordudu et al.

Our systematic review and the underlying evidence base have several notable limitations. A main limitation of the review is the substantial heterogeneity of the sensitivity estimates across studies. High heterogeneity is a common phenomenon in diagnostic test accuracy meta-analyses and is usually attributable to the spectrum effects and methodological shortcomings of the included studies. The subgroup analyses in our review that stratified meta-analyses by sex and by countries with predominantly White, Latin or mixed populations rendered similar estimates for BMI and WC as the overall analyses that also included Asian populations. We would have expected a difference, as WHO recommends lower cut-off values for Asian populations than for White populations^79^. Similarly, removing studies with a high risk of bias had little impact on the results. Nevertheless, many other factors, the impact of which we did not have sufficient data to explore, could have introduced heterogeneity. For example, the age of the participants, which varied widely among the studies, could have had an influence on the results. Without access to individual patient data, we were unable to assess the impact of age. Another potential source is the spectrum of prevalence rates among the studies (5.7^51^ to 95.8%^67^). Studies with a higher disease prevalence most likely include more severely diseased patients, which ultimately leads to a better test performance in this population.

Heterogeneity could also stem from the use of different cut-offs both for determining obesity with the anthropometric measurement tools and for the reference tests in the primary studies. For BMI and WC, the majority of studies adhered to the cut-offs recommended by WHO (BMI: ≥ 30 kg/m^2^, WC: ≥ 88 cm in women and ≥ 102 cm in men)^1,38^. The cut-offs for the reference tests ranged from ≥ 30% to ≥ 43% body fat in women and from ≥ 20% to ≥ 34.6% in men using DXA, with most studies referring to a body fat percentage > 35% in women and > 25% in men as the standards for defining obesity. Even though these cut-offs are widely applied and recommended, it is important to note that they were chosen arbitrarily and lack sound scientific basis^9,80,81^. For example, BMI thresholds have only been based upon visual inspection of the relationship between BMI and mortality^82^. For body fat percentage, there is little evidence supporting the cut-offs due to the lack of studies investigating the relationship between a continuum of body fat percentage values and cardiometabolic disease and mortality^9,80^. In addition to the heterogeneity that is introduced by the application of various cut-off values, the cut-offs themselves remain an issue of debate and should be the focus of future research. However, their validity goes beyond the scope of this review.

The use of various imaging techniques, including DXA, CT, and MRI, could have led to differences in performance estimates. However, imaging techniques are currently considered to be the most accurate tools for body composition analysis because of their ability to accurately discriminate tissues^37,83^. We excluded all studies that used other reference standards, such as bioelectrical impedance analysis or dilution techniques, to increase homogeneity. Also limiting this review is the absence of a “gold standard” to diagnose obesity. Although imaging techniques are generally able to produce good-quality body composition data, they all have their shortcomings. For example, DXA does not differentiate between types of fat. Silver further argues that an accurate body composition analysis measuring excess body fat is insufficient for diagnosing obesity; it would rather need a tool that translates the interplay between body composition and metabolic risks into a new concept of obesity^84^. Nonetheless, until research has elucidated that interplay, obesity assessment relies on body composition data.

Finally, another major limitation of the underlying evidence base is the low methodological quality of the included studies that, together with the inconsistency and heterogeneity of the results, has contributed to the mostly low or very low confidence that we have in the evidence. We rated only six out of the 32 included studies as a low risk of bias. Many studies included convenience sampling, used inappropriate exclusion criteria for study participants, lacked predefined cut-offs for index and reference tests and failed to provide information about the numbers of participants included in the analysis.

The strengths of this systematic review include a comprehensive search strategy in four electronic databases combined with manual reference checking of pertinent research articles and a search for unpublished research studies. The search strategy was peer-reviewed by an additional information specialist. We contacted the authors of the included studies to receive the data of 2 × 2 tables when not reported. During the whole systematic review process, we followed Cochrane methods^34^, which are known to be methodologically sound and rigorous. Despite these efforts, we cannot entirely rule out the possibility that we have overlooked a relevant research study.

The findings of our review should be interpreted cautiously within the context of clinical practice. Thresholds between normal weight, overweight, and obesity are arbitrary and not based on universally agreed upon standards. Our review emphasises the substantial uncertainties that obesity assessment with anthropometric tools bring with them. Methodologically sound studies with appropriate sampling strategies, predefined and valid cut-offs and complete analyses are needed for firm conclusions. Future research should focus on studies that differentiate between age groups, are conducted in a European setting and examine the combined use of anthropometric tools.

## Conclusions

This systematic review shows that BMI and WC have serious limitations for use as obesity screening tools in clinical practice despite their widespread use, and no evidence supports that WHR and WHtR are more suitable than BMI or WC to access body fat. However, due to the lack of alternatives, BMI and WC might still have a role as initial tools for assessing individuals for excess adiposity until new evidence emerges. Nonetheless, one should be aware of the limitations of these tools when interpreting the results. In some clinical circumstances, particularly for BMI or WC results that are borderline between overweight and obesity, it might be useful to conduct further examinations of obesity-related risk factors or to confirm results with imaging techniques (e.g. DXA scans).

## Supplementary information

Supplementary information.

## Data Availability

2 × 2 tables that support the findings of this study are available from the first author (IS) upon reasonable request.
