# Supplementary material for: The performance of anthropometric tools to determine obesity: a systematic review and meta-analysis
Source: Sci Rep. 2020 Jul 29;10:12699. doi: 10.1038/s41598-020-69498-7 (PMC7391719; doi:10.1038/s41598-020-69498-7)
Supplement: Supplementary file 1 — Supplementary information. [file 41598_2020_69498_MOESM1_ESM.pdf]

Supplementary files: The performance of anthropometric tools to determine obesity: a systematic review and meta-analysis

Isolde Sommer[1*], Birgit Teufer[1], Monika Szelag[1], Barbara Nussbaumer-Streit[1], Viktoria Titscher[1], Irma Klerings[1], Gerald Gartlehner[1,2]

[1]Department for Evidence-based Medicine and Evaluation, Danube University Krems, Dr.-Karl-Dorrek-Straße 30, 3500 Krems, Austria

[2]RTI-UNC Evidence-based Practice Center, Research Triangle Institute International, East Cornwallis Road, Post Office Box 12194, Research Triangle Park, North Carolina 27709-2194, USA

Supplementary file 1: Search strategies of the all electronic databases that were searched.

Ovid MEDLINE(R) 1946 to January Week 1 2018, Ovid MEDLINE(R) Epub Ahead of Print January 15, 2018, Ovid MEDLINE(R) In-Process & Other Non-Indexed Citations January 15, 2018, Ovid MEDLINE(R) Daily Update January 15, 2018

Ovid/MEDLINE, 16 Jan 2018

#	Search	Results
1	exp *Overweight/	150701
2	exp *Body Composition/	24297
3	(overweight or obes* or adipos*).ti,kf.	171009
4	((body adj (fat or composition)) or fat distribution).ti,kf.	17989
5	or/1-4	234385
6	*body mass index/ or exp *waist circumference/ or exp *waist-height ratio/ or *waist-hip ratio/	20914
7	(BMI or body mass index).ti,kf.	23802
8	(waist adj2 (hip or height or circumference)).ti,kf.	3273
9	anthropometr*.ti,kf.	10026
10	(BMI or body mass index or (waist adj2 (hip or height or circumference)) or anthropometr*).ab. /freq=5	36349
11	or/6-10	60437
12	5 and 11	28680
13	Tomography, X-Ray Computed/	383882
14	Magnetic Resonance Imaging/	391861
15	Absorptiometry, Photon/	23300
16	Ultrasonography/	181882
17	((comput* adj2 tomograph*) or magnetic resonance imaging or MRI or (dual energy adj2 absorptiometry) or DXA or DEXA or ultrasound or Ultrasonograph*).ti,ab,kf.	864734

18	exp "sensitivity and specificity"/ or predict*.ti. or diagnos*.ti. or accura*.ti. or validat*.ti. or Validation Studies.pt.	1431094
19	exp *Overweight/di, dg or exp *Body Composition/an, ph	9293
20	or/13-19	2522263
21	12 and 20	6368
22	exp animals/ not exp humans/	4816547
23	21 not 22	6356
24	exp age groups/ not exp adult/	1879844
25	23 not 24	5080
26	limit 25 to yr="2000 -Current"	4718
27	(english or german).lg.	26328480
28	26 and 27	4567
29	remove duplicates from 28	4051
30	case report/ or (case? and (report* or series)).ti.	2156933
31	comment/ or editorial/ or letter/ or (editorial or comment or letter or commentary).ti.	1806562
32	30 or 31	3740080
33	29 not 32	3927

Embase.com (Elsevier) 16 Jan 2018

No.	Query	Results
#1	'obesity'/de OR 'abdominal obesity'/de OR 'morbid obesity'/de	376466
#2	'adipose tissue'/exp	147292
#3	'body composition'/de	49592
#4	overweight:ti OR obes*:ti OR adipos*:ti	187611
#5	'body fat':ti OR 'body composition':ti OR 'fat distribution':ti	18730
#6	#1 OR #2 OR #3 OR #4 OR #5	519980

#7	'body mass'/exp/mj OR 'waist circumference'/exp/mj OR 'waist to height ratio'/exp/mj OR 'waist hip ratio'/exp/mj	26139
#8	bmi:ti OR 'body mass index':ti	24239
#9	(waist NEAR/2 (hip OR height OR circumference)):ti	3105
#10	anthropometr*:ti	9227
#11	#7 OR #8 OR #9 OR #10	41042
#12	#6 AND #11	22973
#13	'computer assisted tomography'/de OR 'x-ray computed tomography'/exp	658860
#14	'nuclear magnetic resonance imaging'/exp	772925
#15	'dual energy x ray absorptiometry'/exp	30369
#16	'echography'/exp	695977
#17	'obesity'/dm_di OR 'abdominal obesity'/dm_di OR 'morbid obesity'/dm_di	6589
#18	'adipose tissue'/exp/dm_di	6
#19	((comput* NEAR/2 tomograph*):ti,ab) OR 'magnetic resonance imaging':ti,ab OR mri:ti,ab OR (('dual energy' NEAR/2 absorptiometry):ti,ab) OR dxa:ti,ab OR dexa:ti,ab OR ultrasound:ti,ab OR ultrasonograph*:ti,ab	1071514
#20	'sensitivity and specificity'/exp OR 'validation study'/exp OR 'diagnostic test accuracy study'/exp	369404
#21	predict*:ti OR diagnos*:ti OR accura*:ti OR validat*:ti	1148858
#22	#13 OR #14 OR #15 OR #16 OR #17 OR #18 OR #19 OR #20 OR #21	3241846
#23	#12 AND #22	4785
#24	'animal'/exp NOT 'human'/exp	4973235
#25	#23 NOT #24	4764
#26	'groups by age'/exp NOT 'adult'/exp	2474463
#27	#25 NOT #26	3922
#28	#27 AND [2000-2018]/py	3645
#29	#28 AND ([english]/lim OR [german]/lim)	3512

#30	#29 NOT 'conference abstract'/it	2508
#31	'case report'/exp OR 'case study'/exp OR ((case:ti OR cases:ti) AND (report:ti OR series:ti))	2400105
#32	'editorial'/exp OR 'letter'/exp OR editorial:ti OR comment:ti OR letter:ti OR commentary:ti	1634560
#33	#31 OR #32	3836125
#34	#30 NOT #33	2378

CINAHL (Ebsco) 16 Jan 2018

#	Query	Limiters/Expanders	Results
S1	(MH "Obesity")	Search modes - Find all my search terms	38.073
S2	(MH "Body Composition+")	Search modes - Find all my search terms	11.969
S3	TI (overweight OR obes* OR adipos*) OR SU (overweight OR obes* OR adipos*)	Search modes - Find all my search terms	54.673
S4	TI ("body fat" OR "body composition" or "fat distribution") OR SU ("body fat" OR "body composition" or "fat distribution")	Search modes - Find all my search terms	11.143
S5	S1 OR S2 OR S3 OR S4	Search modes - Find all my search terms	61.911
S6	(MM "Body Mass Index") OR (MM "Waist Circumference") OR (MM "Waist-Hip Ratio")	Search modes - Find all my search terms	5.271
S7	TI (BMI OR "body mass index") OR SU (BMI OR "body mass index")	Search modes - Find all my search terms	42.141
S8	TI (waist N1 (hip OR height OR circumference)) OR SU (waist N1 (hip OR height OR circumference))	Search modes - Find all my search terms	3.343
S9	TI anthropometr*	Search modes - Find all my search terms	1.279
S10	S6 OR S7 OR S8 OR S9	Search modes - Find all my search terms	43.979
S11	S5 AND S10	Search modes - Find all my search terms	17.687

S12	(MH "Tomography, X-Ray Computed") OR (MH "Magnetic Resonance Imaging") OR (MH "Absorptiometry, Photon") OR (MH "Ultrasonography")	Search modes - Find all my search terms	89.261
S13	(MH "Sensitivity and Specificity") OR (MH "Validation Studies")	Search modes - Find all my search terms	71.973
S14	(MH "Obesity/DI/RA/US") OR (MH "Body Composition+/CL")	Search modes - Find all my search terms	1.23
S15	comput* N1 tomograph* OR ("magnetic resonance imaging" OR MRI) OR "dual energy" N1 absorptiometry OR (DXA OR DEXA OR ultrasound OR Ultrasonograph*)	Search modes - Find all my search terms	121.337
S16	TI predict* OR diagnos* OR accura* OR validat*	Search modes - Find all my search terms	114.859
S17	S12 OR S13 OR S14 OR S15 OR S16	Search modes - Find all my search terms	292.649
S18	S11 AND S17	Search modes - Find all my search terms	3.638
S19	(MH "Named Groups by Age+") NOT (MH "Adult+")	Search modes - Find all my search terms	322.549
S20	S18 NOT S19	Search modes - Find all my search terms	2.931
S21	S20	Limiters - Published Date: 20000101-20171231; Language: English, German	2.798
S22	S21	Limiters - Exclude MEDLINE records	541

PubMed 16 Jan 2018

Search	Query	Items found
#1	Search (overweight[Title] OR obes*[Title] OR adipos*[Title])	137850
#2	Search ("body fat"[Title] OR "body composition"[Title] OR "fat distribution"[Title])	14670
#3	Search (#1 OR #2)	150562

#4	Search (BMI[Title] OR "body mass index"[Title])	16285
#5	Search (waist[Title]) AND (hip[Title] OR height[Title] OR circumference[Title])	2221
#6	Search anthropometr*[Title]	7343
#7	Search (#4 OR #5 OR #6)	24956
#8	Search (#3 AND #7)	3483
#9	Search (comput*[tiab] AND tomograph*[tiab]) OR magnetic resonance imaging[tiab] OR MRI[tiab] OR (dual energy[tiab] AND absorptiometry[tiab]) OR DXA[tiab] OR DEXA[tiab] OR ultrasound[tiab] OR Ultrasonograph*[tiab]	787911
#10	Search (predict*[Title] OR diagnos*[Title] OR accura*[Title] OR validat*[Title])	881186
#11	Search (#9 OR #10)	1582294
#12	Search (#8 AND #11)	858
#13	Search pubmednotmedline [sb] OR pmcbook OR (publisher [sb] AND (pubstatusnihms OR pubstatuspmcsd))	2179932
#14	Search (#13 AND #12)	63
#15	Search (#14) AND ("2000"[Date - Publication] : "2017"[Date - Publication])	57
#16	Search #15 AND ("english"[Language] OR "german"[Language])	56

ProQuest Dissertations & Theses Global 16 Jan 2018

Query	Results
all(overweight OR obes* OR adipos* OR "body fat" OR "body composition" OR "fat distribution") AND (ti(BMI OR "body mass index" OR (waist AND (hip OR height OR circumference)) OR anthropometr*) OR su(BMI OR "body mass index" OR (waist AND (hip OR height OR circumference)) OR anthropometr*)) AND all(tomograph* OR "magnetic resonance imaging" OR MRI OR absorptiometry OR DXA OR DEXA OR ultrasound OR Ultrasonograph* OR predict* OR diagnos* OR accura* OR validat*)	
Limits - Date: After December 31 1999; Language: German, English	360

WorldCat dissertations and theses 16 Jan 2018

Search	Query	Results

1	WorldCat dissertations and theses results for: (kw: overweight OR kw: obes* OR kw: adipos* OR kw: body w fat OR kw: body w composition OR kw: fat w distribution) and (ti: BMI OR ti: body w mass w index OR (ti: waist AND (ti: hip OR ti: height OR ti: circumference)) OR ti: anthropometr*) and (kw: tomograph* OR kw: magnetic w resonance w imaging OR kw: MRI OR kw: absorptiometry OR kw: DXA OR kw: DEXA OR kw: ultrasound OR kw: Ultrasonograph* OR kw: predict* OR kw: diagnos* OR kw: accura* OR kw: validat*) and la= "eng"	231
2	(kw: overweight OR kw: obes* OR kw: adipos* OR kw: body w fat OR kw: body w composition OR kw: fat w distribution) and (ti: BMI OR ti: body w mass w index OR (ti: waist AND (ti: hip OR ti: height OR ti: circumference)) OR ti: anthropometr*) and (kw: tomograph* OR kw: magnetic w resonance w imaging OR kw: MRI OR kw: absorptiometry OR kw: DXA OR kw: DEXA OR kw: ultrasound OR kw: Ultrasonograph* OR kw: predict* OR kw: diagnos* OR kw: accura* OR kw: validat*) and yr: 2000-2018 and la= "ger" .	3
3	(kw: overweight OR kw: obes* OR kw: adipos* OR kw: body w fat OR kw: body w composition OR kw: fat w distribution) and (su: BMI OR su: body w mass w index OR (su: waist AND (su: hip OR su: height OR su: circumference)) OR su: anthropometr*) and (kw: tomograph* OR kw: magnetic w resonance w imaging OR kw: MRI OR kw: absorptiometry OR kw: DXA OR kw: DEXA OR kw: ultrasound OR kw: Ultrasonograph* OR kw: predict* OR kw: diagnos* OR kw: accura* OR kw: validat*) and yr: 2000-2018 and la= "eng" .	171
4	(kw: overweight OR kw: obes* OR kw: adipos* OR kw: body w fat OR kw: body w composition OR kw: fat w distribution) and (su: BMI OR su: body w mass w index OR (su: waist AND (su: hip OR su: height OR su: circumference)) OR su: anthropometr*) and (kw: tomograph* OR kw: magnetic w resonance w imaging OR kw: MRI OR kw: absorptiometry OR kw: DXA OR kw: DEXA OR kw: ultrasound OR kw: Ultrasonograph* OR kw: predict* OR kw: diagnos* OR kw: accura* OR kw: validat*) and yr: 2000-2018 and la= "ger"	2
	Total	407

Supplementary file 2: Characteristics of included studies

Table S1: Characteristics of included studies

Author, Year, Country, Funding	Setting	Population<br>Number of participants<br>Mean age in years (SD)<br>Female<br>Ethnicity	Index test and cut-offs for obesity				Reference test and cut-offs for obesity	Prevalence of obesity (%)	Risk of Bias
			BMI (kg/m²)	WC (cm)	WHR	WHtR (cm/m)			
Aschner et al. [57], 2011, Mexico, El Salvador, Venezuela, Colombia, Paraguay, partially financed by pharmaceutical company	Primary care	457<br>Women: 45.9 (16.5)<br>Men: 44.8 (15)<br>60.8%<br>NR	NR	Women: 80-93<br>Men: 90-102	NR	NR	**CT (cm²)**<br>VAT >100	Women: 47.8<br>Men: 55.3	unclear
Batsis et al. [58], 2016, USA, public funding	Community (NHANES)	4,984<br>NR<br>49.0%<br>NR	≥30	Women ≥88<br>Men ≥102	NR	NR	**DXA (% BF)**<br>Women ≥35<br>Men ≥25	Women:<br>≥60 years : 89.1<br>60-69.9 years 91.7<br>70-79.9 years 90.9<br>80+years 80.7<br><br>Men:<br>≥60 years: 87.5<br>60-69.9 years 86.5<br>70-79.9 years 88.4<br>80+years 88.2	low
Blew et al. [73], 2002, USA, public funding	Community -based study	317<br>54.8 (4.8)<br>100.0% (all postmenopausal)<br>87.7% White	≥ 30	NR	NR	NR	**DXA (% BF)**<br>Women: ≥35, 38, 40	NR	high
Carneiro Roriz et al. [43,55,56], 2014, Brazil, public funding	University Hospital and Community	191 -197[1]<br>Adult women (20-59 years): 39.93 (11.35)<br>Elderly women (≥60 years): 73.7 (8.11)<br>Adult men (20-59 years): 39.37 (13.08)<br>Elderly men (≥60 years):	NR	Adult women: 92.3<br>Elderly women: 88.2<br>Adult men: 90.2<br>Elderly men: 92.2	Adult women: 0.87<br>Elderly women: 0.88<br>Adult men: 0.93<br>Elderly men: 0.96	Adult women: 0.59<br>Elderly women: 0.58<br>Adult men: 0.54<br>Elderly men: 0.55	**CT (cm²)**<br>VAT ≥ 130	Adult women: 12.5<br>Elderly women: 42.9<br><br>Adult men: 26.5<br>Elderly men:62.2	unclear

[1] Number of participants included slightly differed between publications

Author, Year, Country, Funding	Setting	Population<br>Number of participants<br>Mean age in years (SD)<br>Female<br>Ethnicity	Index test and cut-offs for obesity				Reference test and cut-offs for obesity	Prevalence of obesity (%)	Risk of Bias
			BMI (kg/m²)	WC (cm)	WHR	WHtR (cm/m)			
		72.19 (8.39)<br>51%<br>NR							
Chen et al. [60], 2006, China, public funding	Hospital	1,122<br>53.0 (4.0)<br>100 %<br>100% Chinese	1)WHO/IASO/ IOTF:<br><br>Obesity >25<br>(2) WGOC:<br><br>Obesity >28	>80	NR	NR	**DXA (% BF)**<br>Obesity >40	NR	low
De Lorenzo et al. [64], 2003, Italy, NR	Community	890<br>Women: 46 (14)<br>Men: 41 (17)<br>61%<br>NR	>30	NR	NR	NR	**DXA (% BF)**<br>Women >35<br>Men >25	Men: 27.2<br>Women: 46.0	high
De Oliveira et al. [62], 2014, Brazil, public funding	Community	300<br>51 (NR)<br>0%<br>NR	NR	**WCNR:**<br>Obesity: 89.4<br>Central obesity: 91.6<br><br>**WCMD:**<br><br>Obesity: 91.4<br>Central obesity: 93.8<br><br>**WCUL:**<br>Obesity: 93.4<br>Central Obesity: 95.6	NR	NR	**DXA (% BF)**<br>20<br>Obesity: 25<br>Central obesity: 75th percentile of %abdominal area fat in the present sample	NR	unclear
Diniz et al. [61], 2016, Brazil, partially financed by pharmaceutical company	Outpatient Clinic	83 (42 Psoriasis, 41 Controls)<br>Psoriasis: 47.0 (NR)<br>Controls: 38 (NR)<br>Psoriasis: 41%<br>Controls: 46%<br>NR	>30	NR	NR	NR	**DXA (% BF)**<br>20-39 years:<br>Women: 39+<br>Men: 26+<br><br>40-59 years:<br>Women: 41+<br>Men: 29+	Total: 50.1<br>PSO: 44.4<br>Control: 58.3	high

Author, Year, Country, Funding	Setting	Population<br>Number of participants<br>Mean age in years (SD)<br>Female<br>Ethnicity	Index test and cut-offs for obesity				Reference test and cut-offs for obesity	Prevalence of obesity (%)	Risk of Bias
			BMI (kg/m²)	WC (cm)	WHR	WHtR (cm/m)			
							60-79 years:<br>Women: 43+<br>Men: 31+		
Donini et al. [48], 2013, Italy, NR	Community and Clinical Rehabilitation Institute	103<br>Women: 41.6 (10.2)<br>Men: 43.8 (8.1)<br>72%<br>NR	>30	NR	NR	NR	**DXA (% BF)**<br>Women ≥35<br>Men ≥ 25	Women and men: 82.5	high
Evans et al. [72], 2006, USA, public funding	Outpatient care	296<br>65.86 (7.52)<br>100%<br>73% White, 27% Black	>30	NR	NR	NR	**DXA (% BF)**<br>≥38	Women: 46.0	high
Goh et al. [51], 2004, Singapore, public funding	Community	1069<br>Women: 48.4 (0.29)<br>Men: 49.4 (0.50)<br>72%<br>97% Chinese	≥ 30	Women ≥ 80.5<br>Men ≥92.5	Women ≥ 0.826<br>Men ≥ 0.94	Women ≥ 0.513<br>Men ≥ 0.55	**DXA (% BF)**<br>Women ≥35<br>Men ≥25	Women: 13.1<br>Men: 5.7	high
Gong et al. [47], 2007, China, public funding	Physical Examination Center in hospital	72<br>48.96 (9.22)<br>39%<br>100% Mongolian	Women 28.4<br>Men: 26.6	Women 93.5<br>Men: 95.0	Women: 0.935<br>Men: 0.925	NR	**MRI (cm²)**<br>VAT ≥110	NR	unclear
Grier et al. [65], 2015, USA, NR	Army	110<br>23 (4.5)<br>0%<br>7% Caucasian, 3% Asian, 8% Black, 9% Hispanic, 6% Other	<21 years: 25.9<br>21–27 years: 26.5<br>28–39 years: 27.2<br>>40 years: 27.5	NR	NR	NR	**DXA (%BF)**<br><21 years: 20<br>21–27 years: 22<br>28–39 years: 24<br>>40 years: 26	Men: 28.2	unclear
Guimaraes et al. [59], 2017, Brazil, public funding	Hospital or primary care	82<br>55.24 (10.77)<br>100% (with rheumatoid arthritis)<br>NR	≥ 23 - 30	≥80 - 86	NR	NR	**DXA (% BF)**<br>39-43	Women: 59.8	unclear
Horie et al. [68], 2006, Japan, NR	Community	516<br>NR<br>0%<br>100% Japanese	≥25	NR	NR	NR	**CT (cm²)**<br>VAT ≥ 100	Men: 47.1	high

Author, Year, Country, Funding	Setting	Population<br>Number of participants<br>Mean age in years (SD)<br>Female<br>Ethnicity	Index test and cut-offs for obesity				Reference test and cut-offs for obesity	Prevalence of obesity (%)	Risk of Bias
			BMI (kg/m²)	WC (cm)	WHR	WHtR (cm/m)			
Jia et al. [49], 2003, China, NR	Outpatient departmen t hospital	690<br>Women <100cm³ VAT: 51.4 (10.0)<br>Women ≥100cm³ VAT: 57.9 (10.1)<br>Men <100cm³ VAT: 54.4 (11.5)<br>Men ≥100cm³ VAT: 59.0 (10.2)<br>56%<br>100% Chinese	23-30	70-105	0.83- 0.97	NR	**MRI (cm²)**<br>VAT ≥ 100	Women: 37.1<br>Men: 43.3	high
Kagawa et al. [71], 2006, Japan, NR	NR	139<br>20.4 (1.3)<br>100%<br>100% Japanese	≥23	NR	NR	NR	**DXA (% BF)**<br>>30	Women: 32.2	unclear
Karlage et al. [54], 2015, USA, public funding	Hospital-based cohort study	1361<br>32.4 (7.7)<br>50%<br>88% White	≥30	NR	NR	≥0.5	**DXA (% BF)**<br>Women ≥30<br>Men ≥25	Women: 84.8<br>Men: 63.1	unclear
Katz et al. [50], 2011, USA, public funding	Clinical Research Center	145 women with systemic lupus erythematosus<br>47.9 (12.2)<br>100%<br>55% White, 10% Hispanic, 15% Black, 12% Asian	≥30<br>≥26.4<br>≥26.8	≥ 88<br>≥ 84.75	≥ 0.85<br>≥ 0.80	NR	**DXA (% BF)**<br>According to Gallagher *<br>(different age and ethnicity categories)	Women: 49.7	high
Marwaha et al. [75], 2014, India, public funding	Community	2347<br>49.1 (18.2)<br>61%<br>100% Indian	>23 - >30	NR	NR	NR	**DXA (% BF)**<br>Women:<br>20–39 years: 39<br>40–59 years: 40<br>Men:<br>20–39 years: 25<br>40–59 years: 28	Women and men: 84.4	unclear

Author, Year, Country, Funding	Setting	Population<br>Number of participants<br>Mean age in years (SD)<br>Female<br>Ethnicity	Index test and cut-offs for obesity				Reference test and cut-offs for obesity	Prevalence of obesity (%)	Risk of Bias
			BMI (kg/m²)	WC (cm)	WHR	WHtR (cm/m)			
Oreopoulos et al. [52,53], 2010, Canada, public funding	University Clinic	140 patients with systolic and/or diastolic chronic heart failure<br>63 (NR)<br>26%<br>90% Caucasian	>30	Women >88<br>Men >102	NR	Women >61.5<br>Men >60.5	**DXA (% BF)**<br>According to Gallagher * (different age and ethnicity categories)	Women and men: 44.3	low
Peterson et al. [66], 2014, USA, public funding	Community (NHANES)	852 adults with impaired mobility<br>Women: 53.09 (1.08)<br>Men: 53.94 (0.68)<br>53%<br>NR	≥ 24-31	NR	NR	NR	**DXA (% BF)**<br>Women ≥ 35<br>Men ≥25	Women: 35.1<br>Men: 29.4	unclear
Pongchaiyakul et al. [70], 2006, Thailand, NR	Community	847<br>Women: 50.5 (15.5)<br>Men: 49.3 (17.2)<br>60%<br>NR	≥ 30	NR	NR	NR	**DXA (% BF)**<br>Women ≥35<br>Men ≥25	Women: 39.4<br>Men: 18.9	low
Pongchaiyakul et al. [63], 2006, Thailand, public funding	Community	436<br>50 (16)<br>Women: 50.6 (15.9)<br>Men: 49.1 (17.1)<br>58%<br>100% Thai		Women:<br>≥ 84<br>≥ 88<br>Men:<br>≥ 93<br>≥ 102	NR	NR	**DXA (% BF)**<br>Women: > 35<br>Men: > 25	Women: 44.0<br>Men: 8.3	low
Rahman et al. [77], 2010, USA, public funding	Hospital	555<br>26.0 (4.0)<br>100%<br>34% White, 29% Black. 37% Hispanic	≥ 25.5-28.7<br>≥ 30	NR	NR	NR	**DXA (% BF)**<br>>35%	Total: 63.1<br>White women: 58.7<br>Black women: 60.4<br>Hispanic women: 69.1	unclear
Ribeiro-Filho et al. [45], 2003, Brazil, public funding	Outpatient Clinic	100<br>50.4 (7.7)<br>100%<br>NR	NR	Women ≥88<br>Men ≥102	Women >0.85<br>Men >1.0	NR	**CT (cm²)**<br>VAT ≥ 130	NR	unclear
Sardinha et al. [76], 2000, Portugal, NR	Community	383<br>60.5 (7.1)<br>100%<br>100%	>30	NR	NR	NR	**DXA (% BF)**<br>>35	Women: 89.3	high

Author, Year, Country, Funding	Setting	Population<br>Number of participants<br>Mean age in years (SD)<br>Female<br>Ethnicity	Index test and cut-offs for obesity				Reference test and cut-offs for obesity	Prevalence of obesity (%)	Risk of Bias
			BMI (kg/m²)	WC (cm)	WHR	WHtR (cm/m)			
Tello-Winniczuk et al. [69], 2017, Mexico, NR	University Hospital	101 patients with rheumatoid arthritis<br>50.54 (12.3)<br>96%<br>NR	≥22<br>≥24<br>≥25<br>≥30	NR	NR	NR	**DXA (% BF)**<br>≥35	Women and men: 92.1	unclear
Temple et al. [74], 2010, Canada, public funding	Outpatient care	46 with mild to moderate intellectual disability<br>33.9 (10.2)<br>46%<br>100% White	>23->30	NR	NR	NR	**DXA (% BF)**<br>Women:<br>20-39 years: 39<br>40–59 years: 40<br>Men:<br>20-39 years: 25<br>40–59 years: 28	Women and men: 62.8	unclear
Vasconcelos et al. [67], 2010, Brazil, public funding	Community	180<br>Women: 67.2 (5.2)<br>Men: 69.14 (5.6)<br>67%<br>NR	>25<br>>27<br>>30	NR	NR	NR	**DXA (% BF)**<br>Women >32<br>Men >25	Women: 95.8<br>Men: 31.6	low
Yang et al. [44,46], 2006, China, public funding	Community	1946 - 1988<br>Women: 26.8 (4.2)<br>Men: 26.9 (3.8)<br>44%<br>100% Chinese	Women:<br>20-30 years: 19.6<br>31-45 years: 21.2<br>Men:<br>20-30 years: 23.5<br>31-45 years: 24.1	Women:<br>20-30 years: 65.8<br>31-45 years: 71.4<br>Men:<br>20-30 years: 78.9<br>31-45 years: 82.4	Women:<br>20-30 years: 0.74<br>31-45 years: 0.79<br>Men:<br>20-30 years: 0.85<br>31-45 years: 0.86	NR	**DXA (% BF)**<br>Women >30<br>Men >25	Women: 26.2<br>Men: 8.6	unclear
Yoon et al. [78], 2015, Korea, public funding	Community (Korea NHANES)	6017<br>NR<br>51%<br>100% Korean	≥25	NR	NR	NR	**DXA (% BF)**<br>Women: ≥ 35<br>Men: ≥ 25	Women: 34.4<br>Men: 22.5	high

% BF, Percentage Body Fat; BMI, Body Mass Index; CT, Computed Tomography; DXA, Dual-energy X-ray Absorptiometry; IASO, International Association for the Study of Obesity; IOTF, International Obesity Task Force; MRI, Magnetic Resonance Imaging; NHANES, National Health and Nutrition Examination Survey; NR, Not Reported; SD, Standard Deviation; VAT, Visceral Adipose Tissue Area; WC, Waist Circumference; WCNR, Waist Circumference at the Narrowest Waist; WCMD, Waist Circumference at the Midpoint between the Superior Border of the Iliac Crest and the Inferior Margin of the Rib; WCUL, Waist Circumference at the Umbilical Line; WHO, World Health Organization; WHR, Waist-Hip Ratio; WHtR, Waist-Height Ratio; WGOC, Working Group on Obesity in China

[*] Gallagher, D *et al.* Healthy percentage body fat ranges: An approach for developing guidelines based on body mass index. *Am.J.Clin.Nutr.* **7,** 694-701 (2000)

Supplementary file 3: Detailed results for sensitivity and specificity for all four anthropometric tools (Tables 1-4)

Table S1: Results for Body Mass Index to assess obesity (27 studies)

Author, year	Women		Men		Women and Men combined	
	**Sensitivity**	**Specificity**	**Sensitivity**	**Specificity**	**Sensitivity**	**Specificity**
Batsis et al., 2016 [58]	≥60 years: 0.77<br>60–69.9 years: 0.80<br>70–79.9 years: 0.79<br>80+ years: 0.66	≥60 years: 0.92<br>60–69.9 years: 0.91<br>70–79.9 years: 0.92<br>80+ years: 0.91	≥60 years: 0.81<br>60–69.9 years: 0.87<br>70–79.9 years: 0.80<br>80+ years: 0.63	≥60 years: 0.81<br>60–69.9 years: 0.76<br>70–79.9 years: 0.87<br>80+ years: 0.87	NR	NR
	≥60 years: 0.39<br>60–69.9 years: 0.45<br>70–79.9 years: 0.38<br>80+ years: 0.23	≥60 years: 0.99<br>60–69.9 years: 1.00<br>70–79.9 years: 1.00<br>80+ years: 1.00	≥60 years: 0.33<br>60–69.9 years: 0.40<br>70–79.9 years: 0.31<br>80+ years: 0.15	≥60 years: 1.00<br>60–69.9 years: 0.99<br>70–79.9 years: 1.00<br>80+ years: 1.00	NR	NR
Blew et al., 2002 [73]	35% BF: 0.20<br>38% BF: 0.26<br>40% BF: 0.29	35% BF: 1.00<br>38% BF: 0.99<br>40% BF: 0.99	NR	NR	NR	NR
Chen et al., 2006 [60]	Obesity ≥34% BF:<br>≥25: 0.75<br>≥28: 0.41	Obesity ≥34% BF:<br>≥25: 0.71<br>≥28: 0.93	NR	NR	NR	NR
	Obesity ≥34% BF:<br>≥25: 0.61<br>≥28: 0.28	Obesity ≥34% BF:<br>≥25: 0.76<br>≥28: 0.94	NR	NR	NR	NR
De Lorenzo et al., 2003 [64]	≥25: 0.70<br>≥30: 0.43	≥25: 0.80<br>≥30: 0.96	≥25: 0.83<br>≥30: 0.60	≥25kg/m2: 0.86<br>≥30kg/m2: 0.93	NR	NR
Diniz et al. 2016 [61]	NR	NR	NR	NR	Total: 0.38<br>Psoriasis: 0.44<br>Controls: 0.33	Total: 0.92<br>Psoriasis: 0.94<br>Controls: 0.90
Donini et al. 2013 [48]	NR	NR	NR	NR	0.48	1.00
Evans et al. 2006 [72]	35% BF:<br>White: 0.47<br>Black: 0.53<br>38% BF:<br>White: 0.60<br>Black: 0.67<br>40% BF:<br>White: 0.71<br>Black: 0.72	35% BF:<br>White: 1.00<br>Black 1.00<br>38% BF:<br>White: 0.97<br>Black: 0.86<br>40% BF:<br>White: 0.93<br>Black: 0.78	NR	NR	NR	NR
Goh et al., 2004 [51]	≥25: 0.61<br>≥30: 0.13	≥25: 0.90<br>≥30: 0.95	≥27: 0.47<br>≥30: 0.07	≥27: 0.89<br>≥30: 0.97	NR	NR
Gong et al., 2007 [47]	0.85	0.86	0.73	0.80	NR	NR

Grier et al. 2015 [65]	NR	NR	≥25: 0.60<br>≥25.9–27.5: 0.77<br>≥28.3: 0.90	≥25: 1.00<br>≥25.9–27.5: 1.00<br>≥28.3: 0.96	NR	NR
Guimaraes et al. 2017 [59]	≥23: 0.92<br>≥25: 0.82<br>≥30: 0.37	≥23: 0.36<br>≥25: 0.58<br>≥30: 0.76	NR	NR	NR	NR
Horie et al. 2006 [68]	NR	NR	≥23: 0.90<br>≥23.5: 0.83<br>≥24: 0.77<br>≥24.5: 0.70<br>≥25: 0.66<br>≥25.5: 0.57<br>≥26: 0.45	≥23: 0.54<br>≥23.5: 0.66<br>≥24: 0.74<br>≥24.5: 0.80<br>≥25: 0.84<br>≥25.5: 0.88<br>≥26: 0.91	NR	NR
Jia et al., 2003 [49]	≥25: 0.86<br>≥26 (optimal threshold): 0.76<br>≥30: 0.35	≥25: 0.62<br>≥26 (optimal threshold): 0.74<br>≥30: 0.95	≥25: 0.81<br>≥26 (optimal threshold): 0.69<br>≥30: 0.24	≥25: 0.70<br>≥26 (optimal threshold): 0.84<br>≥30: 0.99	NR	NR
Kagawa et al. 2006 [71]	≥23: 0.40<br>≥25: 0.09	≥23: 0.95<br>≥25: 0.99	NR	NR	NR	NR
Karlage et al., 2015 [54]	0.47	1.00	0.54	0.94	NR	NR
Katz et al., 2011 [50]	≥26.4: 0.83<br>≥26.8: 0.80<br>≥30: 0.59	≥26.4: 0.93<br>≥26.8: 0.95<br>≥30: 1.00	NR	NR	NR	NR
Marwaha et al. 2014 [75]	≥23: 0.92<br>≥25: 0.89<br>≥30: 0.92	≥23: 0.69<br>≥25: 0.73<br>≥30: 0.75	≥23: 0.78<br>≥25: 0.87<br>≥30: 0.89	≥23: 0.61<br>≥25: 0.65<br>≥30: 0.64	NR	NR
Oreopoulos et al. 2010 & 2011 [52,53]	NR	NR	NR	NR	0.61	0.82
Peterson et al. 2014 [66]	≥25: 0.84<br>≥30: 0.53	≥25: 0.86<br>≥30: 0.98	≥25: 0.90<br>≥30: 0.47	≥25: 0.76<br>≥30: 1.00	NR	NR
Pongchaiyakul et al. 2006 [70]	≥25: 0.70<br>≥30: 0.21	≥25: 0.84<br>≥30: 0.99	≥27: 0.44<br>≥30: 0.13	≥27: 0.95<br>≥30: 0.99	NR	NR
Rahman et al. 2010 [77]	White: 0.48<br>Black: 0.75<br>Hispanic: 0.54<br>Overall: 0.58	White: 1.00<br>Black: 0.97<br>Hispanic: 0.98<br>Overall: 0.99	NR	NR	NR	NR
Sardinha et al. 2000 [76]	≥25.5: 0.74<br>≥30: 0.27	≥25.5: 0.92<br>≥30: 1.00	NR	NR	NR	NR

Tello-Winniczuk et al. 2017 [69]	NR	NR	NR	NR	22: (>35% AF): 0.97<br>24: (>40% BF): 0.90<br>25: (>35% BF): 0.86<br>30: (>35% AF): 0.43<br>30: (>40% BF): 0.46<br>30: (>35% BF): 0.42	22: (>35% AF) 0.84<br>24: (>40% BF) 0.75<br>25: (>35% BF) 0.39<br>30: (>35% AF): 1.00<br>30:(>40% BF): 0.89<br>30: (>35% BF): 1.00
Temple et al. 2010 [74]	NR	NR	NR	NR	≥25: 0.86<br>≥26 (optimal threshold): 0.86<br>≥30: 0.57	≥25: 0.71<br>≥26 (optimal threshold): 0.88<br>≥30: 1.00
Vasconcelos et al. 2010 [67]	≥25: 0.76<br>≥27: 0.56<br>≥30: 0.28	≥25: 1.00<br>≥27: 1.00<br>≥30: 1.00	≥25: 0.5<br>≥27: 0.74<br>≥30: 0.32	≥25: 0.40<br>≥27: 0.73<br>≥30: 0.98	NR	NR
Yang et al. 2006 & Li et al., 2008 [44,46]	20-30-years: 0.83<br>31-45 years: 0.86	20-30-years: 0.57<br>31-45 years: 0.66	20-30-years: 0.85<br>31-45 years: 0.85	20-30-years: 0.85<br>31-45 years: 0.76	NR	NR
Yoon et al. 2015 [78]	Total: 0.72<br>20-29 years: 0.85<br>30-39 years: 0.72<br>40-49 years: 0.61<br>50-59 years: 0.66<br>60-69 years: 0.74	Total: 0.84<br>20-29 years: 0.87<br>30-39 years: 0.87<br>40-49 years: 0.86<br>50-59 years: 0.78<br>60-69 years: 0.81	Total: 0.56<br>20-29 years: 0.64<br>30-39 years: 0.51<br>40-49 years: 0.40<br>50-59 years: 0.39<br>60-69 years: 0.85	Total: 89<br>20-29 years: 0.93<br>30-39 years: 0.84<br>40-49 years: 0.93<br>50-59 years: 0.87<br>60-69 years: 0.88	NR	NR

AF, Abdominal Fat; BF, Body Fat; NR, Not Reporte;

Table S2: Results for Waist Circumference to assess obesity (14 studies)

**Author, year**	**Women**		**Men**		**Women and Men combined**	
	**Sensitivity**	**Specificity**	**Sensitivity**	**Specificity**	**Sensitivity**	**Specificity**
Aschner, et al. 2011 [57]	80: 0.92<br>81: 0.92<br>82: 0.90<br>83: 0.89<br>84: 0.87<br>85: 0.86<br>86: 0.84<br>87: 0.82<br>88: 0.82<br>89: 0.81<br>90: 0.79<br>91: 0.76<br>92: 0.73<br>93: 0.72	80: 0.35<br>81: 0.37<br>82: 0.40<br>83: 0.40<br>84: 0.43<br>85: 0.46<br>86: 0.49<br>87: 0.55<br>88: 0.59<br>89: 0.63<br>90: 0.68<br>91: 0.72<br>92: 0.75<br>93: 0.74	90: 0.98<br>91: 0.98<br>92: 0.95<br>93: 0.95<br>94: 0.90<br>95: 0.85<br>96: 0.79<br>97: 0.77<br>98: 0.75<br>99: 0.71<br>100: 0.66<br>101: 0.60<br>102: 0.55	90: 0.54<br>91: 0.59<br>92: 0.64<br>93: 0.75<br>94: 0.80<br>95: 0.83<br>96: 0.88<br>97: 0.90<br>98: 0.90<br>99: 0.90<br>100: 0.90<br>101: 0.93<br>102: 0.94	NR	NR
Batsis et al., 2016 [58]	All: 0.82<br>≥60years: 0.81<br>60–69.9years: 0.81	All: 0.81<br>≥60years: 0.86<br>60–69.9years: 0.90	All: 0.74<br>≥60 years: 0.60<br>60–69.9 years: 0.65	All: 0.89<br>≥60 years: 0.96<br>60–69.9 years: 0.96	NR	NR

	70–79.9years: 0.82<br>80+ years: 0.77	70–79.9years: 0.86<br>80+ years: 0.81	70–79.9 years: 0.60<br>80+ years: 0.44	70–79.9 years: 0.93<br>80+ years: 1.00		
Carneiro Roriz et al., 2011 [43]	Adult women: 0.83<br>Elderly women: 0.76	Adult women: 0.81<br>Elderly women: 0.69	Adult men: 0.87<br>Elderly men: 0.79	Adult men: 0.86<br>Elderly men: 0.78	NR	NR
Chen et al., 2006 [60]	≥34% BF: 0.75<br>≥37% BF: 0.84<br>≥40% BF: 0.88	≥34% BF: 0.75<br>≥37% BF: 0.65<br>≥40% BF: 0.56	NR	NR	NR	NR
De Oliveira, et al. 2014 [62]	NR	NR	Obesity<br>WCNR: 0.85<br>WCMD: 0.84<br>WCUL: 0.85	Obesity<br>WCNR: 0.84<br>WCMD: 0.82<br>WCUL: 0.85	NR	NR
			Central Obesity<br>WCNR: 0.80<br>WCMD: 0.80<br>WCUL: 0.82	Obesity<br>WCNR: 0.80<br>WCMD: 0.80<br>WCUL: 0.81		
Goh et al., 2004 [51]	0.48	0.90	0.47	0.89	NR	NR
Gong et al., 2007 [47]	0.85	0.73	0.77	0.65	NR	NR
Guimaraes et al. 2017 [59]	80: 0.96<br>82: 0.92<br>86: 0.82	80: 0.18<br>82: 0.27<br>86: 0.36	NR	NR	NR	NR
Jia et al., 2003 [49]	70: 1.00<br>75: 1.00<br>80: 0.98<br>85: 0.95<br>90: 0.79<br>95: 0.13<br>100: 0.07<br>105: 0.03	70: 0.10<br>75: 0.21<br>80: 0.34<br>85: 0.57<br>90: 0.74<br>95: 0.87<br>100: 0.93<br>105: 0.97	70: 1.00<br>75: 1.00<br>80: 1.00<br>85: 0.96<br>90: 0.78<br>95: 0.47<br>100: 0.27<br>105: 0.15	70: 0.08<br>75: 0.18<br>80: 0.34<br>85: 0.58<br>90: 0.83<br>95: 0.94<br>100: 0.99<br>105:0.99	NR	NR
Katz et al., 2011 [50]	84.75: 0.85<br>88: 0.74	84.75: 0.89<br>88: 0.90	NR	NR	NR	NR
Oreopoulos et al. 2010 & 2011 [52,53]	NR	NR	NR	NR	0.90	0.60
Pongchaiyakul et al. 2006 [63]	84: 0.67<br>88: 0.49	84: 0.87<br>88: 0.95	93: 0.47<br>102: 0.20	93: 0.98<br>102: 1.00	NR	NR
Ribeiro-Filho et al. 2003 [45]	0.83	0.61	NR	NR	NR	NR
Yang et al. 2006 & Li et al., 2008 [44,46]	20-30-years: 0.78<br>31-45 years: 0.78	20-30-years: 0.65<br>31-45 years: 0.78	20-30-years: 0.94<br>31-45 years: 0.85	20-30-years: 0.76<br>31-45 years: 0.73	NR	NR

BF, Body Fat; NR, Not Reported; WCNR, Waist Circumference at the Narrowest Waist; WCMD, Waist Circumference at the Midpoint between the Superior Border of the Iliac Crest and the Inferior Margin of the Rib; WCUL, Waist Circumference at the Umbilical Line

Table S3: Results for Waist to Hip Ratio to assess obesity (7 studies)

Author, year	Women		Men	
	Sensitivity	Specificity	Sensitivity	Specificity
Jia et al., 2003 [49]	0.69	0.65	0.79	0.72
Goh et al., 2004 [51]	0.34	0.82	0.47	0.91
Carneiro Roriz et al., 2011 [43]	Adult women: 0.83<br>Elderly women: 0.67	Adult women: 0.72<br>Elderly women: 0.52	Adult men: 0.87<br>Elderly men: 0.83	Adult men: 0.83<br>Elderly men: 0.83
Katz et al., 2011 [50]	Original cut-off: 0.42<br>Revised cut-off: 0.69	Original cut-off: 0.85<br>Revised cut-off: 0.67	NR	NR
Gong et al., 2007 [47]	0.92	0.46	0.82	0.25
Ribeiro-Filho et al. 2003 [45]	0.79	0.64	NR	NR
Yang et al. 2006 & Li et al., 2008 [44,46]	20-30-years: 0.74<br>31-45 years: 0.69	20-30-years: 0.65<br>31-45 years: 0.79	20-30-years: 0.82<br>31-45 years: 0.89	20-30-years: 0.78<br>31-45 years: 0.64

NR, Not Reported;

Table S4: Results for Waist to Height Ratio to assess obesity (4 studies)

Author, year	Women		Men		Women and Men combined	
	Sensitivity	Specificity	Sensitivity	Specificity	Sensitivity	Specificity
Karlage et al., 2015 [54]	0.70	0.95	0.87	0.71	NR	NR
Goh et al., 2004 [51]	0.51	0.90	0.47	0.89	NR	NR
Carneiro Roriz et al., 2014 [55]	Adult women: 0.83<br>Elderly women: 0.81	Adult women: 0.84<br>Elderly women: 0.79	Adult men: 0.87<br>Elderly men: 0.86	Adults men: 0.86<br>Eldery men: 0.82	NR	NR
Oreopoulos et al., 2010 & Oreopoulos et al., 2011 [52,53]	NR	NR	NR	NR	0.77	0.77

NR, Not Reported;

Supplementary file 4: Figures of subgroup analyses based on ethnicity, showing summary ROC curves (Figure S1) and paired forest plots for BMI and WC (Figure S2)

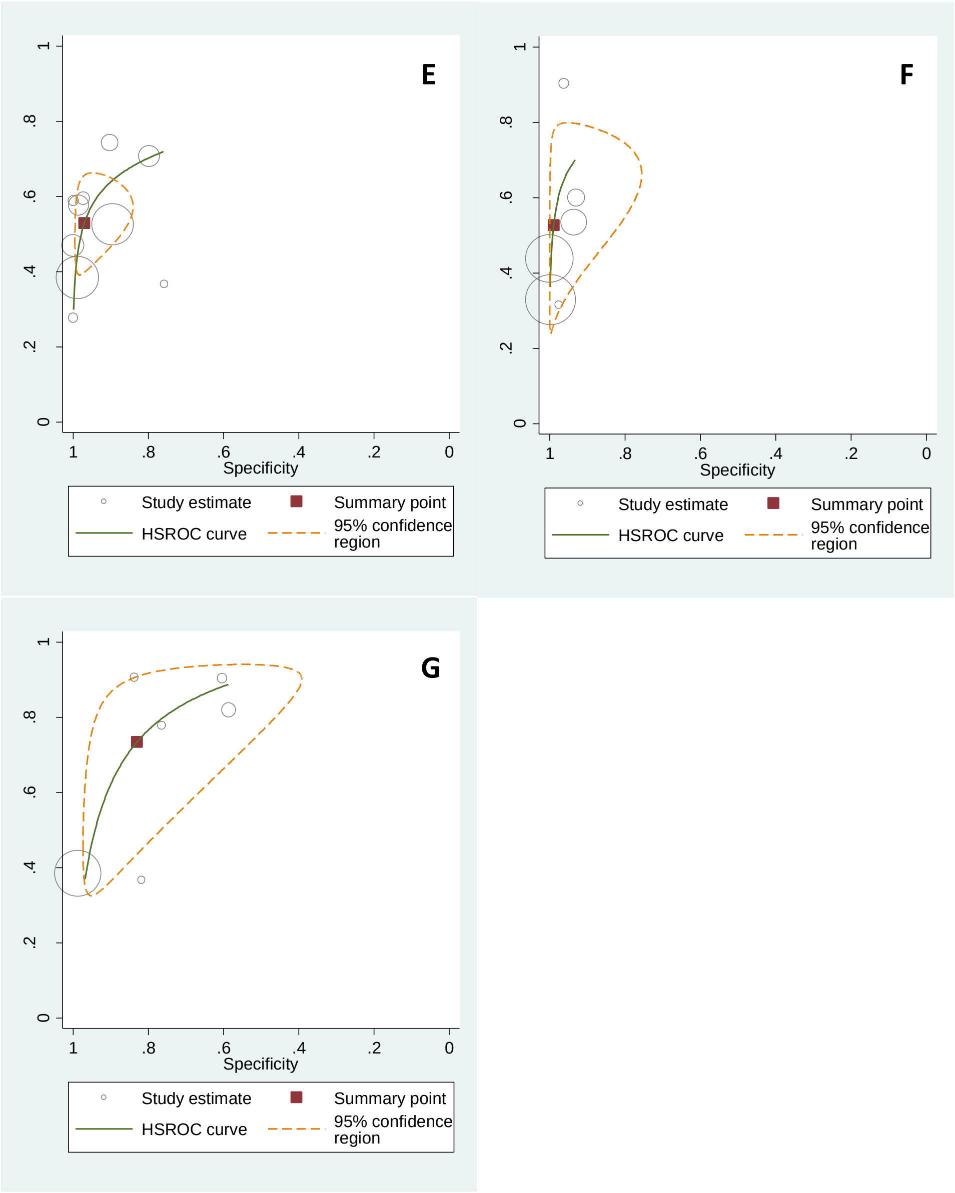

Figure S1 Summary ROC Curve including summary point for Body Mass Index (BMI) in E) Women of White, Latin or Mixed Ethnicity (sensitivity: 52.9% [95% CI 43.8% to 61.9%]; specificity: 97.0% [95% CI 90.8% to 99.1%]), F) Men of White, Latin or Mixed Ethnicity (sensitivity: 52.8% [95%CI 36.4% to 68.6%],specificity: 98.9% [95%CI 93.8% to 99.8%]), and for Waist Circumference (WC) in G) White and Latin Women (sensitivity: 73.4% [95% CI 52.5% to 87.4%]; specificity: 83.0% [95% CI 62.7% to 93.4%])

BMI: White, Latin, and Mixed Women

Study	TP	FP	FN	TN	Sensitivity (95% CI)	Specificity (95% CI)
Batsis 2016	841	3	1345	264	0.38 [0.36, 0.41]	0.99 [0.97, 1.00]
De Lorenzo 2003	194	65	80	257	0.71 [0.65, 0.76]	0.80 [0.75, 0.84]
Evans 2006	62	3	42	110	0.60 [0.50, 0.69]	0.97 [0.92, 0.99]
Guimaraes 2017	18	8	31	25	0.37 [0.23, 0.52]	0.76 [0.58, 0.89]
Karlage 2015	273	0	309	104	0.47 [0.43, 0.51]	1.00 [0.97, 1.00]
Katz 2011	43	0	30	72	0.59 [0.47, 0.70]	1.00 [0.95, 1.00]
Peterson 2014	443	24	400	1536	0.53 [0.49, 0.56]	0.98 [0.98, 0.99]
Rahman2010	202	3	148	202	0.58 [0.52, 0.63]	0.99 [0.96, 1.00]
Sardinha 2000	254	4	88	37	0.74 [0.69, 0.79]	0.90 [0.77, 0.97]
Vasconcelos 2010	32	0	83	5	0.28 [0.20, 0.37]	1.00 [0.48, 1.00]

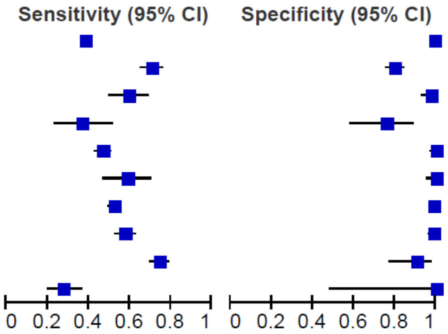

WC White and Latin Women

Study	TP	FP	FN	TN	Sensitivity (95% CI)	Specificity (95% CI)
Aschner 2011	109	60	24	85	0.82 [0.74, 0.88]	0.59 [0.50, 0.67]
Batsis 2016	2118	47	513	275	0.81 [0.79, 0.82]	0.85 [0.81, 0.89]
Carneiro Roriz 2011	21	17	6	55	0.78 [0.58, 0.91]	0.76 [0.65, 0.86]
Guimaraes 2017	18	6	31	27	0.37 [0.23, 0.52]	0.82 [0.65, 0.93]
Katz 2011	53	7	19	66	0.74 [0.62, 0.83]	0.90 [0.81, 0.96]

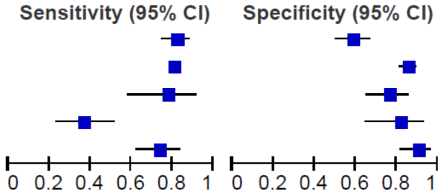

BMI: White, Latin and Mixed Men

Study	TP	FP	FN	TN	Sensitivity (95% CI)	Specificity (95% CI)
Batsis 2016	729	1	1486	315	0.33 [0.31, 0.35]	1.00 [0.98, 1.00]
De Lorenzo 2003	48	15	32	199	0.60 [0.48, 0.71]	0.93 [0.89, 0.96]
Grier 2015	28	3	3	76	0.90 [0.74, 0.98]	0.96 [0.89, 0.99]
Karlage 2015	228	16	198	233	0.54 [0.49, 0.58]	0.94 [0.90, 0.96]
Peterson 2014	321	0	362	1639	0.47 [0.43, 0.51]	1.00 [1.00, 1.00]
Vasconcelos 2010	6	1	13	40	0.32 [0.13, 0.57]	0.98 [0.87, 1.00]

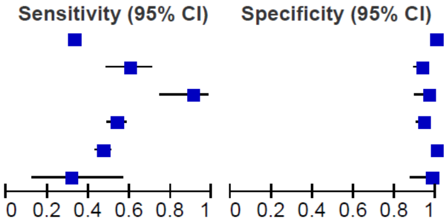

Figure S2: Paired Forest-plots of Sensitivity and Specificity for Body Mass Index (BMI) and Waist Circumference (WC): Subgroup Analysis for Ethnicity; data not weighted
